# Supplementary material for: Strategies and techniques for quality control and semantic enrichment with multimodal data: a case study in colorectal cancer with eHDPrep
Source: Gigascience. 2023 May 12;12:giad030. doi: 10.1093/gigascience/giad030 (PMC10176503; doi:10.1093/gigascience/giad030)
Supplement: giad030_Supplemental_Tables [file giad030_supplemental_tables.zip › eHDPrep_supplement.pdf]

SUPPLEMENTARY MATERIAL

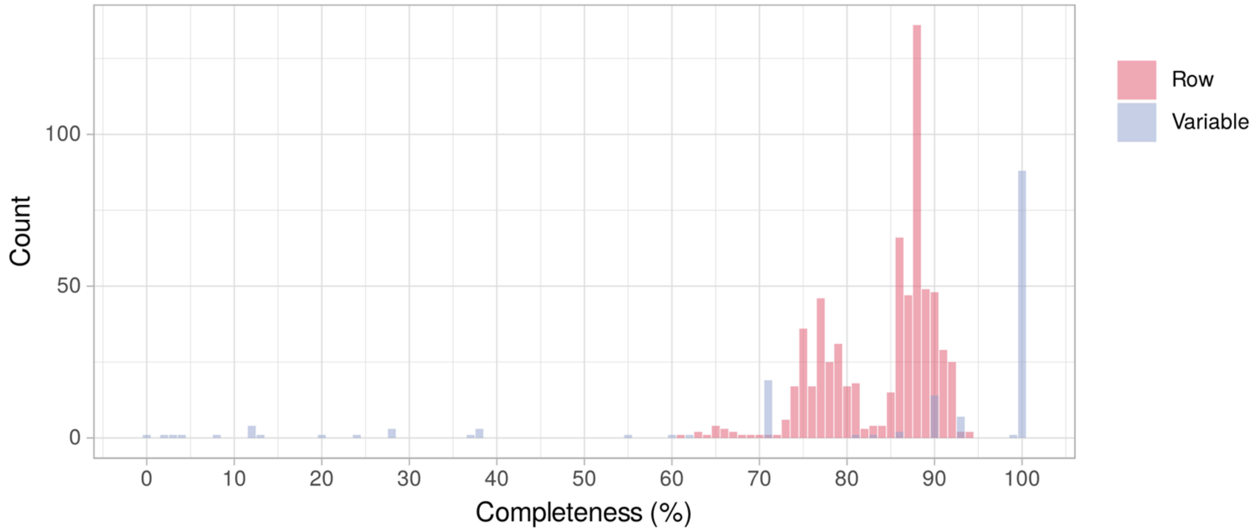

Supplementary Figure S1: Completeness in Colo-661. *This bar plot summarises patient record (red) and variable (lilac) completeness in the unprocessed Colo-661 dataset. Patient records (red) were between 61% and 94% complete while variable completeness (blue) ranged from 0% to 100%.*

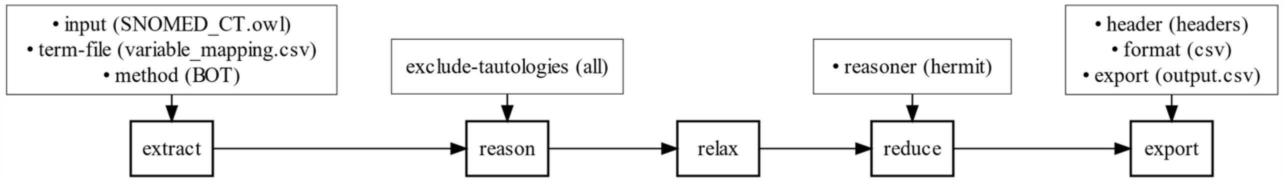

Supplementary Figure S2: Preparation of SNOMED CT for semantic enrichment. *ROBOT commands are joined with horizontal arrows. The arguments are shown above their corresponding commands, joined with a vertical arrow with applied parameters displayed in brackets. The "extract" command was used to subset the input ontology. The "BOT" method subset all terms in the ontology to entities mapped to Colo-661 variables plus all super-classes and inter-relations between super-classes. The "reason" command was used to logically validate and automatically classify the ontology using the reasoner "hermit" with all tautologies removed. The "relax" command was used to relax Equivalence axioms to weaker SubClassOf axioms which is suitable for semantic enrichment. The "reduce" command removed redundant SubClassOf axioms using the "hermit" reasoner. Finally, the "export" command exported the ontology as a comma separated values for import into R.*

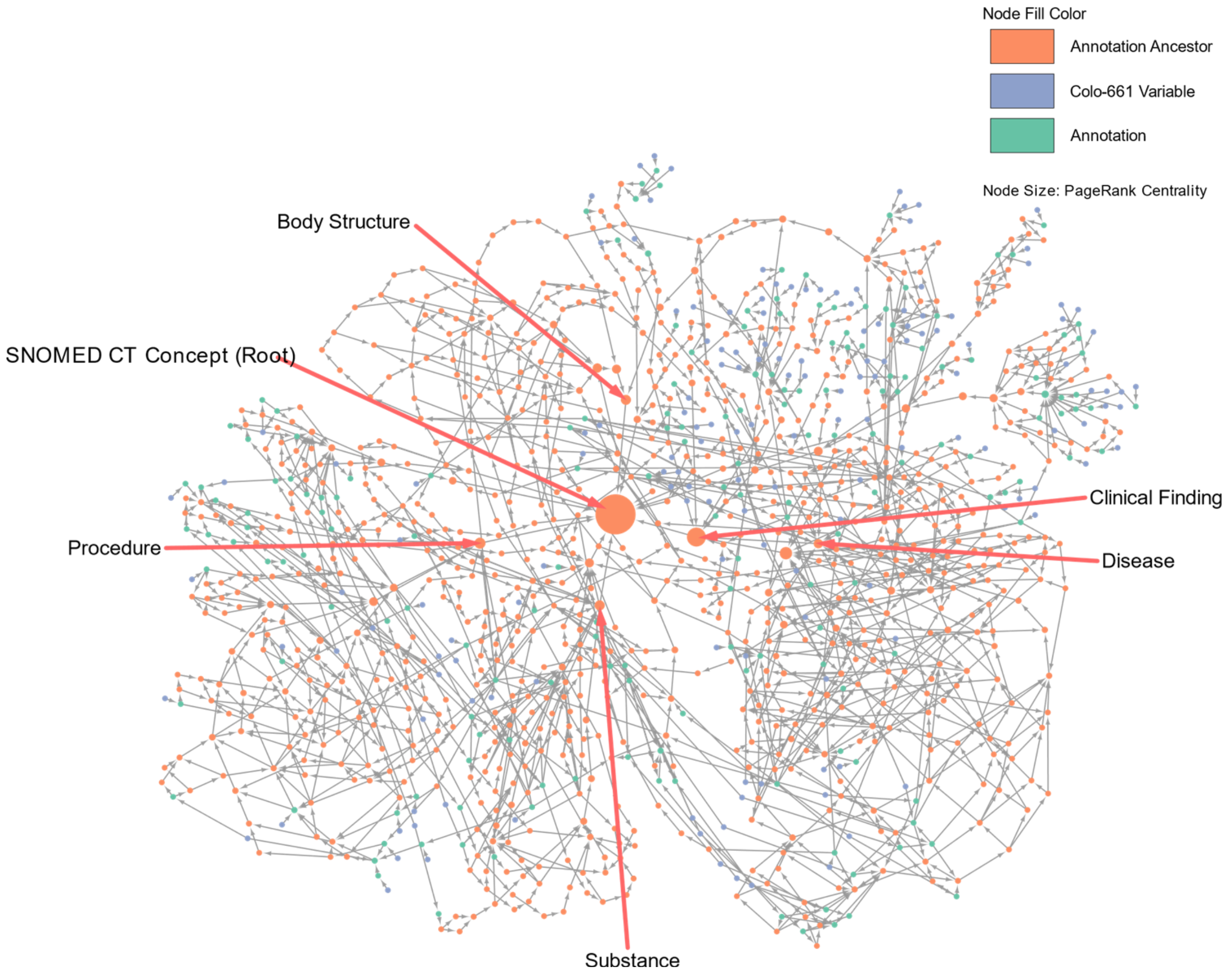

Supplementary Figure S3: SNOMED CT annotation network. *Network nodes represent Colo-661 variables (blue), SNOMED CT terms mapped to Colo-661 variables (green) and their ancestor ontology terms (orange). Node size is proportional to PageRank centrality* [71]. *The large nodes are highly central, representing domains within the network, some of these are labelled with their SNOMED CT term names (red arrows).*

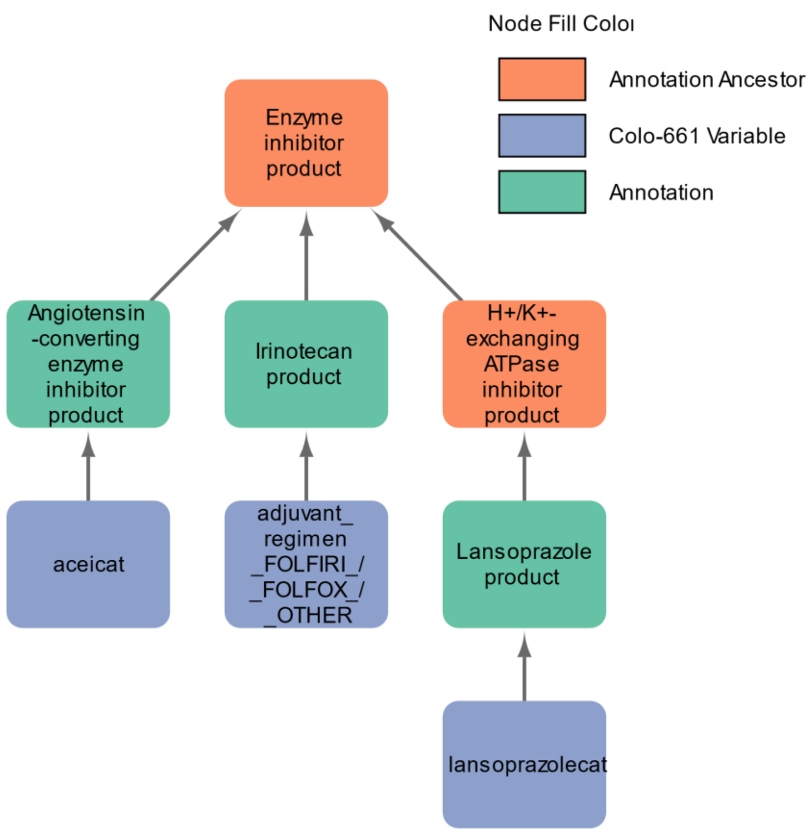

Supplementary Figure S4: Example of a Most Informative Common Ancestor (MICA) term 'Enzyme inhibitor product'. *Variables describing two medications ('aceicat' and 'lansoprazolecat') and an adjuvant regimen were semantically linked through the SNOMED CT term 'Enzyme inhibitor product'. Therefore, semantic enrichment of Colo-661 identified a degree of functional similarity across distinct treatment regimes.*

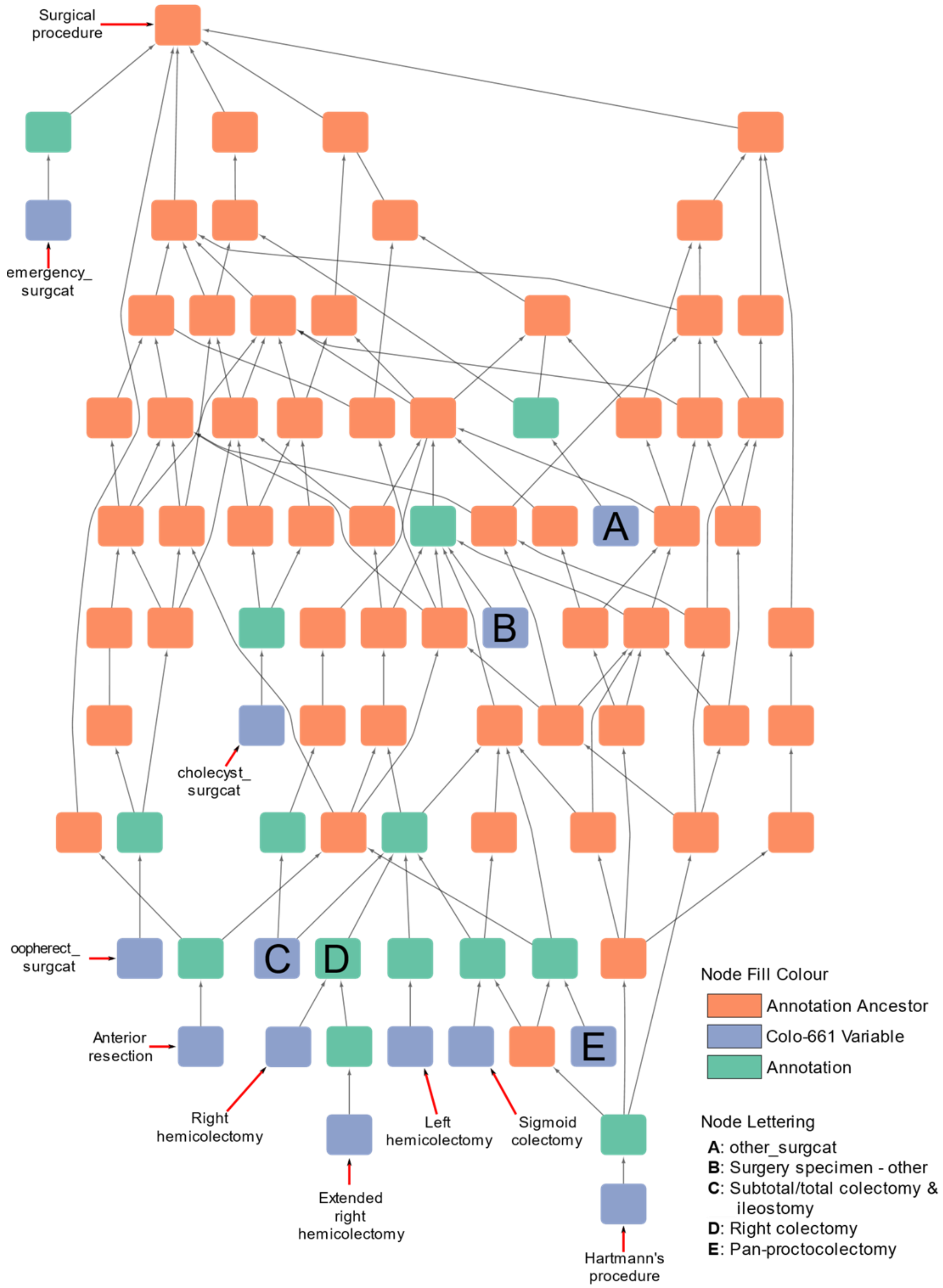

Supplementary Figure S5: SNOMED CT Network for Most Informative Common Ancestor (MICA) term 'Surgical procedure'. *Colo-661 variables describing the procedure used to excise the primary tumour, or describing other*

operations, and the emergency status of the patient's operation are semantically linked via the 'Surgical procedure' MICA. This MICA encompasses a relatively large number of variables (n=13) aggregating information across a range of surgical procedures that could be useful in later analyses. This network also includes several other MICAs, corresponding to smaller groupings of Colo-661 variables, such as node D (Right colectomy), which aggregates the variables 'Right hemicolectomy' and 'Extended right hemicolectomy'.

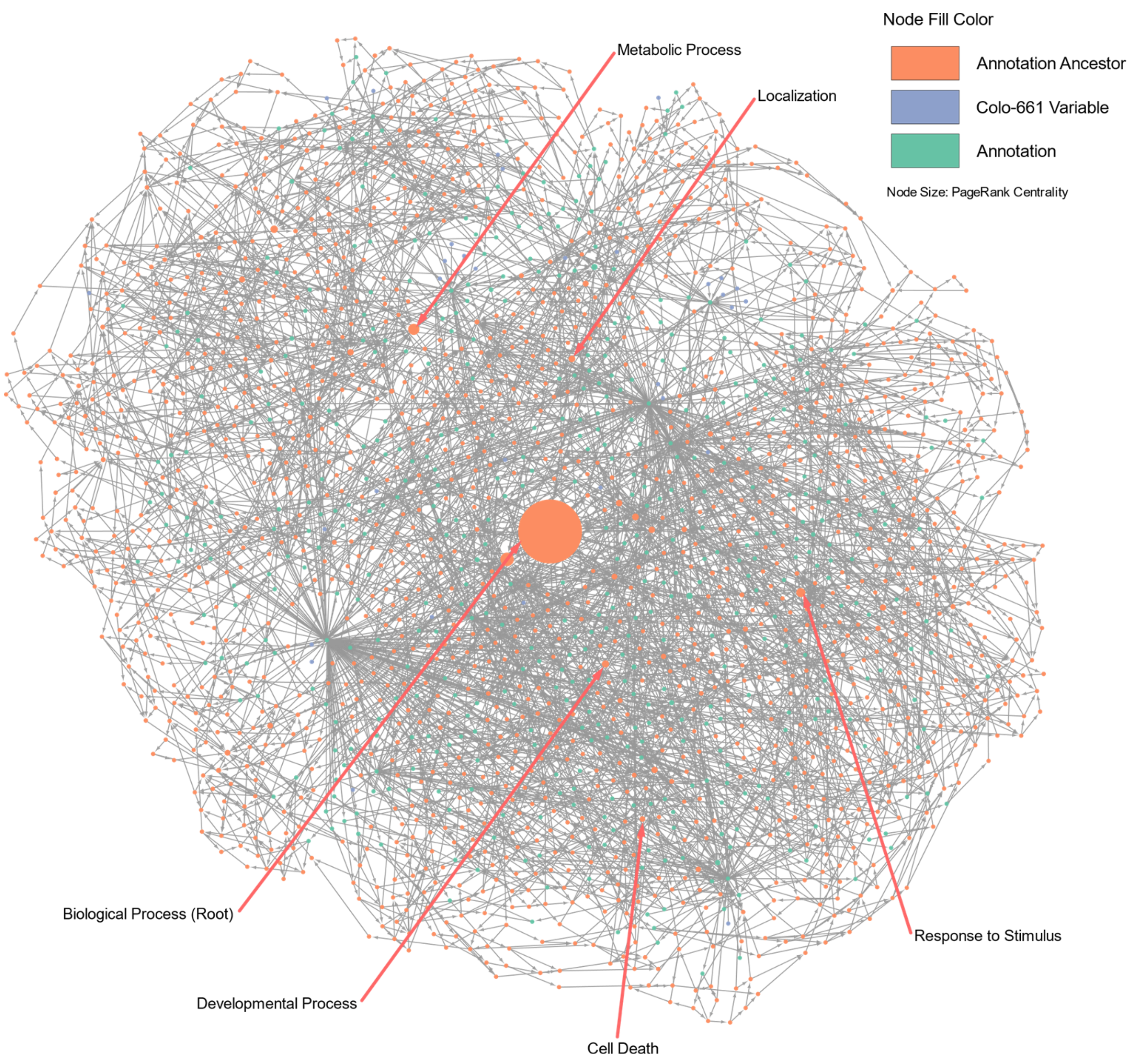

Supplementary Figure S6: The Colo-661 Gene Ontology (GO) Biological Process annotation network. *Network nodes show Colo-661 variables (blue), their mapped genes (green) with associated GO terms (green), and ancestor terms (orange). Node size is proportional to PageRank centrality* [71]. *Larger nodes have high PageRank centrality, represent domains within the network and some of these are labelled (red arrows) with their names from GO.*

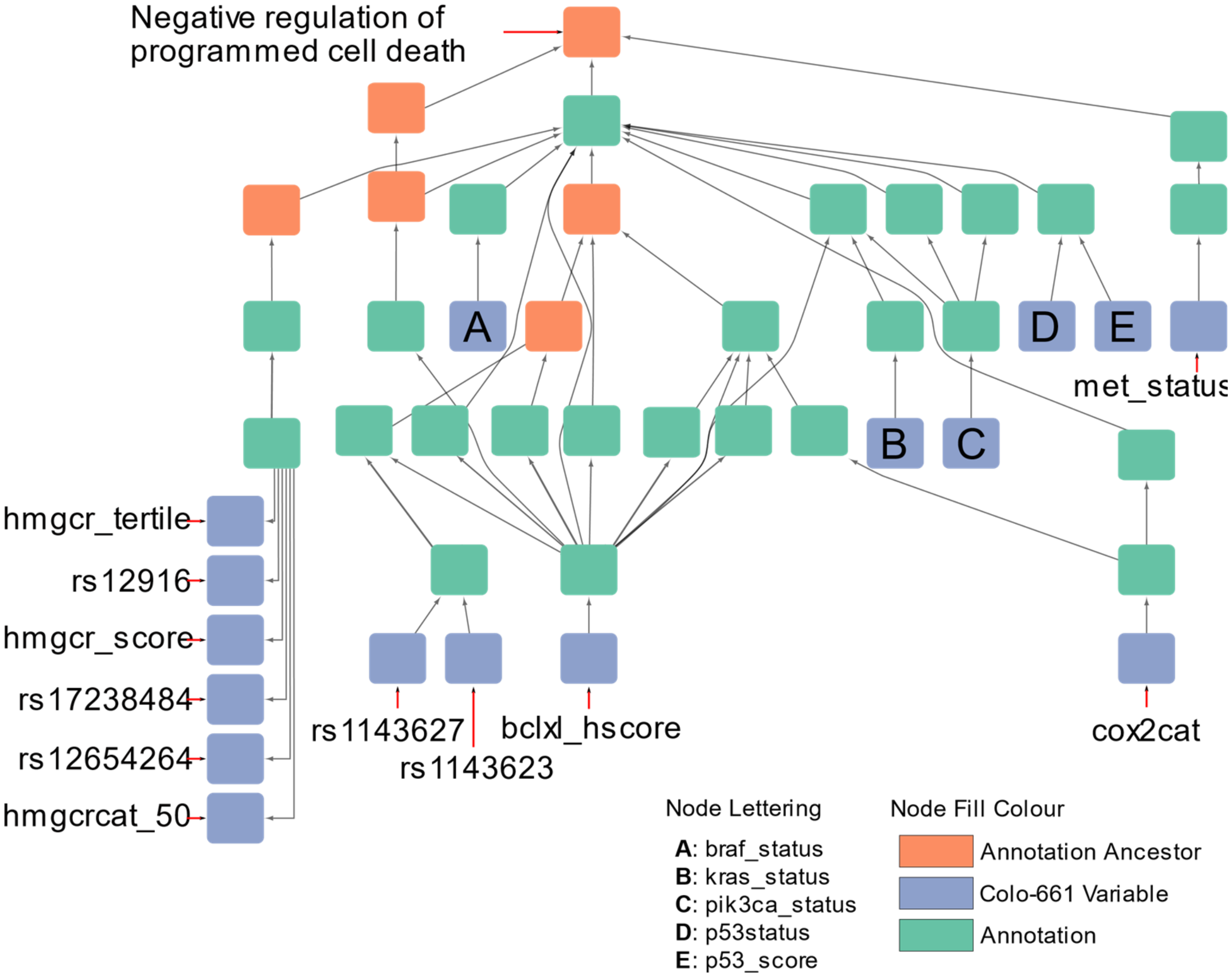

Supplementary Figure S7: Gene Ontology Biological Process Network for the Most Informative Common Ancestor (MICA) term 'Negative regulation of programmed cell death'. *Network nodes show Colo-661 variables (blue), their mapped genes (green) with associated GO terms (green), and their ancestor terms (orange). The 'Negative regulation of programmed cell death' MICA describes an important step in the progression of many cancers* [48], *where cells can evade signals that lead to cell death. Additionally, the Figure exemplifies aggregation of variables from different data modalities. For example, 'hmgcr_tertile' and 'rs12916' at the bottom left of the figure are immunohistochemical and single nucleotide polymorphism variables, respectively.*

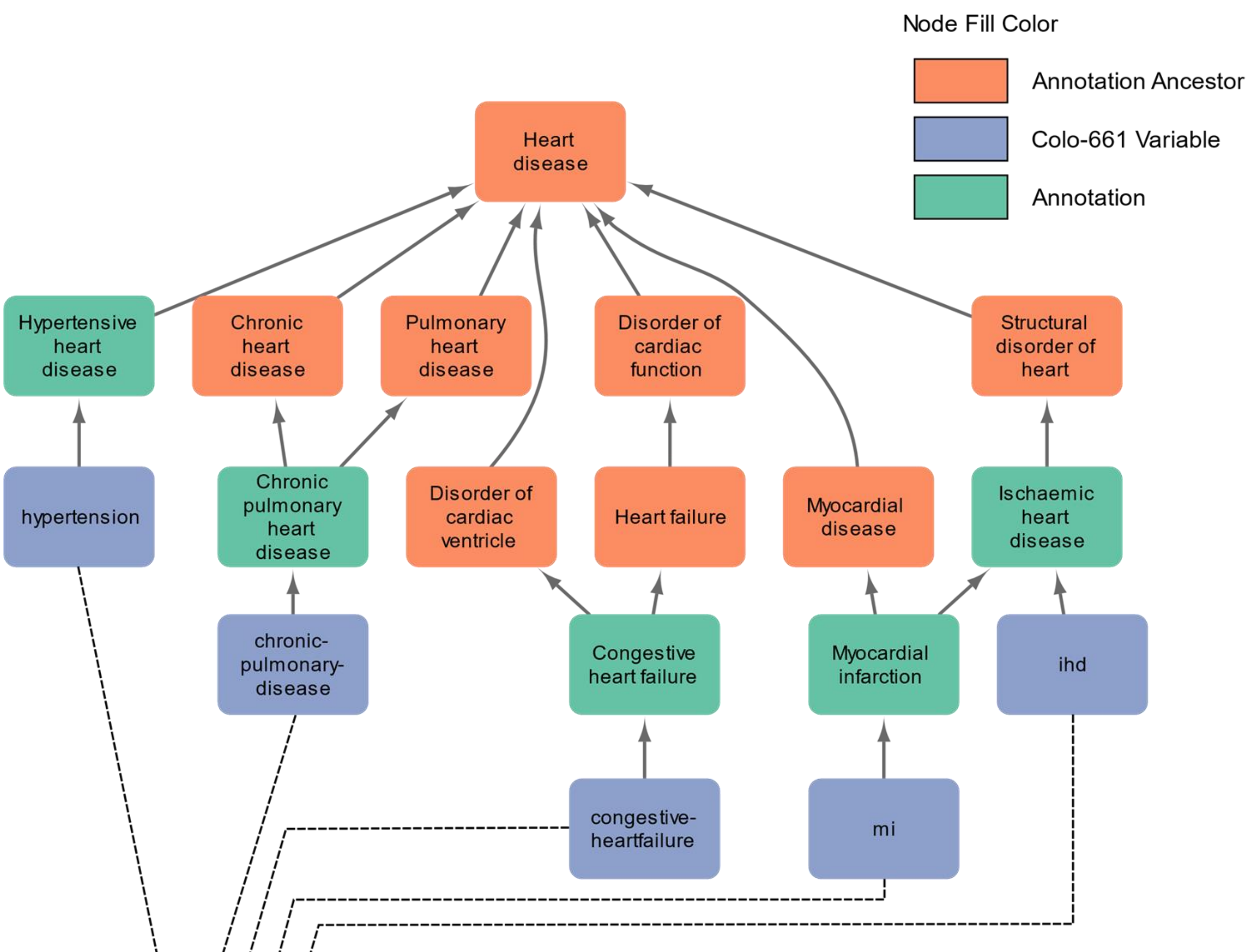

Patient ID							Heart_Disease_Sum	Heart_Disease_Minimum	Heart_Disease_Maximum	Heart_Disease_Average	Heart_Disease_Product
A	1	1	1	0	0	→	3	0	1	0.6	0
B	1	0	1	1	1	→	4	0	1	0.8	0
C	1	0	1	0	0	→	2	0	1	0.4	0
D	1	1	1	1	0	→	4	0	1	0.8	0
E	1	0	1	1	1	→	4	0	1	0.8	0
F	0	0	1	0	1	→	2	0	1	0.4	0
G	1	0	0	1	1	→	3	0	1	0.6	0
H	0	1	1	0	1	→	3	0	1	0.6	0
I	1	1	0	1	1	→	4	0	1	0.8	0
J	1	0	1	0	1	→	3	0	1	0.6	0

Supplementary Figure S8: Worked example of aggregation in semantic enrichment. *Five variables from Colo-661 in the network (blue) were mapped to entities in SNOMED CT that share 'Heart disease' as their Most Informative Common Ancestor (MICA). These five variables therefore constitute a "set" and are selected from example synthetic data containing these variables' names (table, bottom-left) and are aggregated row-wise. The aggregated variables (table, bottom-right) are appended to the synthetic dataset, labelled with the MICA's name and the corresponding aggregation function.*

Supplementary Table S1: List of Colo-661 variables and ontological mappings. [Provided as a separate file]. *Variables which were added, preserved or removed can be identified by the "Presence Post-QC" column. The user-defined variable modality is recorded in the "Modality" column. Data classes, as encoded in R, are given in the "Data Types" columns. Ranges of values are shown in "Value Range in Post-QC Dataset"; no range is given if a variable was removed from the dataset during QC. Our mapping(s) are provided in the columns "Mapped Ontology" and "Ontological / Gene Mapping". Variables mapped to the GO require an initial mapping to a gene, shown here, with Gene:GO term mappings detailed in Supplementary Table S3.*

Supplementary Table S2: Internal consistency checks performed on Colo-661. [Provided as a separate file]. *Tests between variables both containing numeric values were performed using the logical operator in 'Logical Test' with the format '[Variable A] [Operator] [Variable B]'. Between variables containing categories, values in 'Variable B Boundaries' were tested to only be present given the corresponding values in 'Variable A Boundaries'. Tests between numeric and categorical values were similarly compared with numeric (inclusive) ranges denoted by colon-separated values.*

Supplementary Table S3: List of genes mapped to Colo-661 variables and the mapped GO terms. [Provided as a separate file]. *GO terms are separated by ";". Mappings between genes and GO terms were sourced from Ensembl.*

Supplementary Table S4: Redundancy between meta-variables and a consituent variable. *Two of five meta-variables derived from minimum and product aggregations of 'bisoprolol_cat' and 'betablocker_cat' (semantically linked by the MICA: 'beta adrenergic receptor blocking agent therapy') were fully redundant with 'bisoprolol_cat'. The table describes the observed row-wise combinations of values across the constituent variables and the two meta-variables which were redundant with 'bisoprolol_cat'. While the value of 'betablocker_cat' differed from values of the meta-variables shown, the value of 'bisoprolol_cat' did not which led to the observed redundancy. 'bisoprolol_cat' and 'betablocker_cat' described if patients were prescribed bisoprolol and beta blockers, respectively.*

bisoprolol_cat	betablocker_ca t	'Minimum' meta-variable	'Product' meta-variable
0	1	0	0
0	0	0	0
1	1	1	1
