# Supplementary material for: Strategies and techniques for quality control and semantic enrichment with multimodal data: a case study in colorectal cancer with eHDPrep
Source: Gigascience. 2023 May 12;12:giad030. doi: 10.1093/gigascience/giad030 (PMC10176503; doi:10.1093/gigascience/giad030)
Supplement: giad030_Response_to_Reviewer_Comments_Original_Submission [file giad030_response_to_reviewer_comments_original_submission.pdf]

Author's Response To Reviewer Comments

Close

Please see PDF in submission 'response_to_reviewers.pdf' Additionally, the Supplementary Data File (zipfile) has been sent by email.

Close
