# Supplementary material for: Strategies and techniques for quality control and semantic enrichment with multimodal data: a case study in colorectal cancer with eHDPrep
Source: Gigascience. 2023 May 12;12:giad030. doi: 10.1093/gigascience/giad030 (PMC10176503; doi:10.1093/gigascience/giad030)
Supplement: giad030_Reviewer_1_Report_Original_Submission — Janna Hastings -- 11/17/2022 Reviewed [file giad030_reviewer_1_report_original_submission.pdf]

Reviewer Report

Title: Strategies and Techniques for Quality Control and Semantic Enrichment with Multimodal Data: A Case Study in Colorectal Cancer with eHDPrep

Version: Original Submission **Date:** 11/17/2022

Reviewer name: Janna Hastings

Reviewer Comments to Author:

The manuscript describes a toolkit for the automated semantic enrichment and quality control of electronic health data using ontologies. This is a much needed utility that will add value to electronic data sharing and re-use for many different purposes including the development of machine learning for medical applications and personalised medicine. Overall the manuscript is well written and the functionality offered by the toolkit is well thought out and motivated. The internal consistency checks and the use of ontology-based information content to semantically aggregate variables into more informative meta-variables are particularly welcome functions. However, I recommend that the description of the tool functionality be clarified in some points, and the evaluation could be strengthened.page 6-7, internal consistency: 1. How should the user specify semantic dependencies between variable pairs? Would it not be helpful to use a standard format for this specification to enable interoperability and re-use of such specifications? 2. Should the specification of semantic relationships between variables not be linked to the knowledge from the ontologies? Ontologies are able to represent many different types of logical relationships between classes, which make them ideal for then serving as a standard and interoperable format for specifying this type of constraint. Rules are another promising standard approach for logic-based knowledge representation.Page 11, figure 4 a: I think it would be informative for evaluating the operation of the tool if the heatmap of variable missingness after application of the tool could also be illustrated beside the current Fig 4a.Page 13, ontology preparation: The paragraph describes what the authors have done to prepare ontologies for use with the tool. Is this preparation procedure also necessary for users to follow when they use the eHDPrep tool? How can alternative ontologies be incorporated (which may be useful for other domains)?Evaluation: The biggest shortcoming of the presented manuscript is that the evaluation is limited to the application of the tool to one dataset and subsequent manual evaluation of the outcome by one group, the study authors. The results as presented are positive, but there is a significant risk that the tool performs well on this task, as assessed by these study authors, but then fails to generalise to other tasks and datasets that future users might wish to use it with. To mitigate against this challenge, it would be optimal if somewhat more independent methods could be found for evaluating the performance of the different aspects of the tool. One approach could a rigorous comparison of this tool's performance against the performance of other tools that have similar functionality, e.g. comparison of the semantic aggregation function with other tools that find and recommend MICAs. An alternative approach might be to apply the tool to an additional dataset for which a group outside of the study authors would be prepared to provide an independent evaluation.

Level of Interest

Please indicate how interesting you found the manuscript: Choose an item.

Quality of Written English

Please indicate the quality of language in the manuscript: Choose an item.

Declaration of Competing Interests

Please complete a declaration of competing interests, considering the following questions:

- Have you in the past five years received reimbursements, fees, funding, or salary from an organisation that may in any way gain or lose financially from the publication of this manuscript, either now or in the future?
- Do you hold any stocks or shares in an organisation that may in any way gain or lose financially from the publication of this manuscript, either now or in the future?
- Do you hold or are you currently applying for any patents relating to the content of the manuscript?
- Have you received reimbursements, fees, funding, or salary from an organization that holds or has applied for patents relating to the content of the manuscript?
- Do you have any other financial competing interests?
- Do you have any non-financial competing interests in relation to this paper?

If you can answer no to all of the above, write 'I declare that I have no competing interests' below. If your reply is yes to any, please give details below.

I declare that I have no competing interests.

I agree to the open peer review policy of the journal. I understand that my name will be included on my report to the authors and, if the manuscript is accepted for publication, my named report including any attachments I upload will be posted on the website along with the authors' responses. I agree for my report to be made available under an Open Access Creative Commons CC-BY license (http://creativecommons.org/licenses/by/4.0/). I understand that any comments which I do not wish to be included in my named report can be included as confidential comments to the editors, which will not be published.

Choose an item.

To further support our reviewers, we have joined with Publons, where you can gain additional credit to further highlight your hard work (see: https://publons.com/journal/530/gigascience). On publication of this paper, your review will be automatically added to Publons, you can then choose whether or not to claim your Publons credit. I understand this statement.

Yes Choose an item.
