# Supplementary material for: Strategies and techniques for quality control and semantic enrichment with multimodal data: a case study in colorectal cancer with eHDPrep
Source: Gigascience. 2023 May 12;12:giad030. doi: 10.1093/gigascience/giad030 (PMC10176503; doi:10.1093/gigascience/giad030)
Supplement: giad030_Reviewer_2_Report_Original_Submission — Hugo Leroux -- 11/21/2022 Reviewed [file giad030_reviewer_2_report_original_submission.pdf]

**Reviewer Report**

**Title: Strategies and Techniques for Quality Control and Semantic Enrichment with Multimodal Data: A Case Study in Colorectal Cancer with eHDPrep**

**Version: Original Submission** **Date:** 11/21/2022

**Reviewer name: Hugo Leroux**

**Reviewer Comments to Author:**

This well-written paper describes techniques for semantically-enriching clinical data pertaining to colorectal cancer diagnosis.It describes an R-based tool, eHDPrep, to extract the data, which is subsequently cleaned, actioned for missing and erroneous values, encoded and enriched semantically using SNOMED CT and the GO, and ultimately exported after having undergone some QC.The paper is well-written and the methods really well-explained, for which the authors should be commended.I only have a few comments for the authors:1. It is not clear to me how, in the discussion on page 14, the authors have dealt with the issue of representing negative findings and missing values, as described within their enrichment outcomes section.2. In the "Ontology Preparation" section, the authors describe how they have taken both the SNOMED CT terminology and performed some transformations to OWL and conversion to CSV format before mapping the Colo-661 variables to it. They don't however discuss the challenges that such an approach entails. The authors might consider perusing through this article (https://doi.org/10.1186/s13326-018-0191-z), which addresses many of the challenges relating to ontology matching3. Please insert an additional ")" when stating the "Equations", e.g. page 6: "... zero entropy [27] (Equation (1)) ...", also , page 13

**Level of Interest**

Please indicate how interesting you found the manuscript: Choose an item.

**Quality of Written English**

Please indicate the quality of language in the manuscript: Choose an item.

**Declaration of Competing Interests**

Please complete a declaration of competing interests, considering the following questions:

- Have you in the past five years received reimbursements, fees, funding, or salary from an organisation that may in any way gain or lose financially from the publication of this manuscript, either now or in the future?
- Do you hold any stocks or shares in an organisation that may in any way gain or lose financially from the publication of this manuscript, either now or in the future?

- Do you hold or are you currently applying for any patents relating to the content of the manuscript?
- Have you received reimbursements, fees, funding, or salary from an organization that holds or has applied for patents relating to the content of the manuscript?
- Do you have any other financial competing interests?
- Do you have any non-financial competing interests in relation to this paper?

If you can answer no to all of the above, write 'I declare that I have no competing interests' below. If your reply is yes to any, please give details below.

I declare that I have no competing interests

I agree to the open peer review policy of the journal. I understand that my name will be included on my report to the authors and, if the manuscript is accepted for publication, my named report including any attachments I upload will be posted on the website along with the authors' responses. I agree for my report to be made available under an Open Access Creative Commons CC-BY license (http://creativecommons.org/licenses/by/4.0/). I understand that any comments which I do not wish to be included in my named report can be included as confidential comments to the editors, which will not be published.

Choose an item.

To further support our reviewers, we have joined with Publons, where you can gain additional credit to further highlight your hard work (see: https://publons.com/journal/530/gigascience). On publication of this paper, your review will be automatically added to Publons, you can then choose whether or not to claim your Publons credit. I understand this statement.

Yes Choose an item.
